# Obituary—Sir Chas. Bell, M. Ossan, M. Devergie, M. Fricke, M. J. Fontenelle

**Published:** 1842-06-04

**Authors:** 


*Obituary.* Sir Charles Bell, the distinguished physiologist and surgeon died on the 29th of April, at Hallon Hall, near Worcester, England.

M. Ossan, president of the Medico-Chirurgical Society of Berlin, and editor of the "Journal of Practical Medicine," died at Berlin, on the 11th of January, in the 55th year of his age.

M. Devergie, author of a "Treatise on the Venereal Disease," died at Paris on the 13th of March last.

We have also to record the deaths of M. Fricke, of Hamburg, and of M. J. Fontenelle, of Paris.

